# Routine Diagnostics Confirm Novel Neurodevelopmental Disorders

**DOI:** 10.3390/genes13122305

**Published:** 2022-12-07

**Authors:** Robin-Tobias Jauss, Sophia Schließke, Rami Abou Jamra

**Affiliations:** Institute of Human Genetics, University of Leipzig Medical Center, Philipp-Rosenthal-Straße 55, 04103 Leipzig, Germany

**Keywords:** routine diagnostics, neurodevelopmental disorder, epilepsy, exome sequencing, gene–disorder association

## Abstract

Routine diagnostics is biased towards genes and variants with satisfactory evidence, but rare disorders with only little confirmation of their pathogenicity might be missed. Many of these genes can, however, be considered relevant, although they may have less evidence because they lack OMIM entries or comprise only a small number of publicly available variants from one or a few studies. Here, we present 89 individuals harbouring variants in 77 genes for which only a small amount of public evidence on their clinical significance is available but which we still found to be relevant enough to be reported in routine diagnostics. For 21 genes, we present case reports that confirm the lack or provisionality of OMIM associations (*ATP6V0A1, CNTN2, GABRD, NCKAP1, RHEB, TCF7L2*), broaden the phenotypic spectrum (*CC2D1A, KCTD17, YAP1*) or substantially strengthen the confirmation of genes with limited evidence in the medical literature (*ADARB1, AP2M1, BCKDK, BCORL1, CARS2, FBXO38, GABRB1, KAT8, PRKD1, RAB11B, RUSC2, ZNF142*). Routine diagnostics can provide valuable information on disease associations and support for genes without requiring tremendous research efforts. Thus, our results validate and delineate gene–disorder associations with the aim of motivating clinicians and scientists in diagnostic departments to provide additional evidence via publicly available databases or by publishing short case reports.

## 1. Introduction

Exome sequencing has become increasingly relevant for routine diagnostics, providing timely and cost-efficient diagnosis for patients with suspected genetic disorders [1]. With this diagnostic approach, not only the detected variants but also the genes themselves have to be evaluated regarding their gene–disease associations (GDAs). Several efforts have been made to provide lists of diagnostically relevant genes to be implemented in routine exome sequencing, such as PanelApp, GenCC and the MorbidGenes Panel [2,3,4]. However, not all of these disease-associated genes are supported at the same level. The GDA for *BRCA1*, for example, is supported by evidence from hundreds of researchers and continuously evolving research efforts. Searching for “BRCA1” in PubMed results in more than 1500 publications from 2021 alone, with an increasing number over the past several years. On the other hand, there are genes with reported GDAs that are only supported by initial cohorts and/or sporadic case reports. These genes are nevertheless relevant for routine diagnostics, as they represent novel disorders which might be missed in approaches focusing on genes with highly confident GDAs, as, for example, in panel diagnostics.

Reporting variants in genes with limited evidence is a potential burden in routine diagnostics, and diagnostic departments may refrain from reporting them. However, this hampers the accumulation of information in databases, which in turn provide evidence on genes and their clinical relevance. This means that a subset of rare disorders or disorders with vague phenotypes are less likely to be reported, even if the geneticists in question have the information and are able to report it. The patients and their families are eventually not well-informed, and the diagnostic odyssey or suboptimal management will continue. Routine diagnostics can in fact provide valuable phenotypic and genotypic information for these genes.

With a retrospective analysis of our cohort of nearly 10,000 analyses, we aimed to confirm present but weak GDAs, expand the evidence and causative variants on rare Mendelian genes, and confirm hitherto provisional OMIM associations. We also suggest a few points and recommendations for handling variants in rare disorders to be implemented in routine diagnostics, inviting diagnostic labs to contribute to the accumulation of knowledge on these disorders.

## 2. Materials and Methods

### 2.1. Study Cohort

The study cohort consisted of all next-generation sequencing (NGS) analyses at the Institute of Human Genetics of the University Hospital and Clinics Leipzig in the years 2016 to 2021. This in-house dataset comprised 9449 analyses and 7519 individuals, with 2716 reported variants in 927 genes (see also the ClinVar submissions of the Institute of Human Genetics in Leipzig https://www.ncbi.nlm.nih.gov/clinvar/submitters/506086/, accessed on 7 December 2022). Of these, we retrospectively evaluated all genes with limited gene–disorder association (GDA) evidence which were nevertheless reported to the referring physicians and the patients. We defined limited GDA evidence as having fewer than ten pathogenic/likely pathogenic variants in ClinVar [5] and HGMD [6] combined, as of December 2021. Written informed consent was obtained from all individuals presented in this study. The ages and symptoms of these individuals are given based on the time of the last clinical examination.

### 2.2. Methods and Statistics

Table operations and plots were performed in R v.4.1.2 [7] and ggplot2 [8]. The number of (likely) pathogenic variants in ClinVar and HGMD were counted for each of these genes with a custom R Script. A cut-off of 10 (likely) pathogenic variants was applied and resulted in a preliminary list of genes potentially worth publishing as a case report (Supplementary Table S1). For these genes, literature searches using PubMed and OMIM were performed manually by a medical doctor and an experienced postdoc in biology. After these literature searches, we evaluated whether a detailed case report for this gene would be worthwhile. The criteria for a case report’s being worthwhile are described in Table 1.

## 3. Results

**Figure 1:**
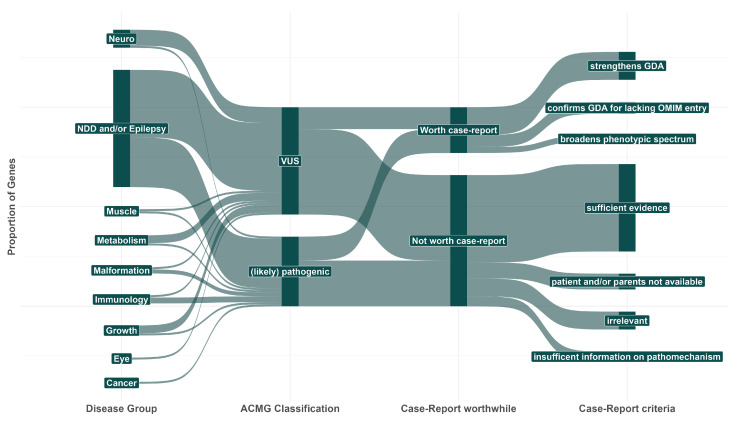
Sankey diagram representing the proportions of disease groups associated with the cases, the ACMG variant classification and the final conclusion about whether a case report on the variant is worthwhile. Most cases were associated with neurodevelopmental delay with or without seizures. Of these, most identified variants were classified with uncertain significance, where a report was often worthwhile based on the criteria mentioned at the right end of the flowchart. NDD: Neurodevelopmental delay. VUS: Variant of uncertain significance. GDA: Gene–disease association.

In 89 individuals from our study cohort, we reported 77 genes to referring physicians with 10 or fewer pathogenic variants in ClinVar and HGMD (Supplementary Table S1). On average, the genes harboured 6.4 (likely) pathogenic variants. There were 66 genes associated with a definite OMIM phenotype and 11 that lacked an entry or were pending validation, as indicated by question marks or curly brackets. Of the 89 cases, we considered 27 as unambiguously solved, 43 as possibly solved with respect to some uncertainty and five as partially solved, i.e., only explaining some of the symptoms. In total, 35 variants were classified as pathogenic or likely pathogenic. The majority of the cases were associated with neurodevelopmental delay, epilepsy or both (Figure 1).

For 56 genes, we did not consider a case report worthwhile based on the criteria described in Table 1. Of these, 35 genes already had sufficient evidence on their GDAs based on several reports or larger cohorts. Five genes had insufficient evidence regarding their pathomechanisms compared to our identified variant, seven genes could not be followed up because the patient or the parents were not available for further analyses, and nine genes turned out to be irrelevant after retrospective analyses. This was mainly due to outdated evidence, i.e., putative phenotypic associations could not be confirmed after re-evaluation or were found to be irrelevant after subsequent Trio-Exome sequencing revealed more convincing de novo variants (for details, see Supplementary Table S1).

**Figure 2:**
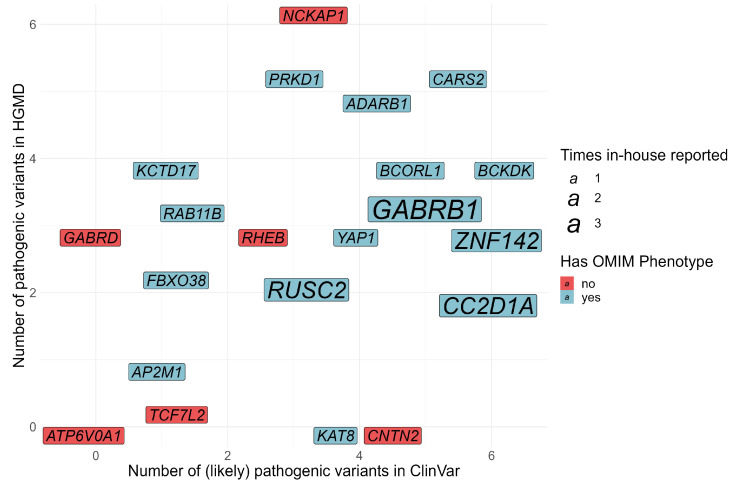
Genes with limited evidence on their pathogenicity reported in our routine diagnostics. Genes are plotted based on the number of pathogenic variants in ClinVar (*x*-axis), HGMD (*y*-axis) and OMIM (colour), as of December 2021. For details, see Table 2.

The remaining 21 genes support current gene–disease associations, add valuable genetic or phenotypic information, or confirm phenotypic associations for genes lacking OMIM entries. An overview of these genes is illustrated in Figure 2 and Table 2.

In the following sections, we present a selection of genes contributing most to the validation of gene–disease associations or expansion of the phenotypic spectrum.

### 3.1. Genes without an OMIM Entry

#### 3.1.1. *NCKAP1*

A possible association between *NCKAP1* (HGNC:7666) and intellectual disability has already been proposed in 2017, while subsequent studies have further supplemented evidence for this gene–disease association through the collection of larger cohorts [9,10,11,12]. However, until now, there has been no OMIM association between *NCKAP1* and the phenotypes of intellectual disability and autism, as proposed by the abovementioned studies.

In a 9 years-old boy presenting with global developmental delay, focal seizures, autistic behaviour, epicanthus, narrow face, anteverted nose and high palate, we identified the heterozygous de novo variant NM_205842.3:c.3366_3369del, p.(Tyr1122*). The variant lies in the last exon of the gene in an area with expected escape of nonsense-mediated decay. It could therefore only be classified as a variant of uncertain significance with the applied criteria PVS1_MOD, PS2_MOD and PM2_SUP [13]. However, the phenotypic overlap and the fact that the variant was identified as de novo constituted sufficient evidence for disease causality, so that the case could be considered solved, further validating *NCKAP1* as a causative gene for neurodevelopmental disorders and autism.

#### 3.1.2. *ATP6V0A1*

As of December 2021, *ATP6V0A1* (HGNC:865) lacked a phenotype association in OMIM (*192130). In the meantime, two studies provided phenotypic details of individuals with identified variants in this gene, resulting in two OMIM entries: autosomal dominant developmental and epileptic encephalopathy (MIM #619970) and autosomal recessive neurodevelopmental disorder with epilepsy and brain atrophy (MIM #619971) [14,15]. Both studies suggested de novo missense variants as being causative for developmental and epileptic encephalopathy, while individuals harbouring missense variants in trans with loss-of-function variants present with the recessive neurodevelopmental disorder with epilepsy and brain atrophy.

Here, we report a 6 years-old boy with global developmental delay, seizures since the age of four weeks, microcephaly, hypotonia and macrosomia. Single exome sequencing in August 2020 was unremarkable but revealed a candidate variant NM_001130021.3:c.2219G>A, p.(Arg740Gln) in the *ATP6V0A1* gene, which was unpublished at the time of initial evaluation. Re-evaluation in February 2022 revealed emerging evidence of variants in this gene being linked to an epilepsy phenotype, and subsequent segregation analyses identified this variant as not maternally inherited, though the father was not available for genetic testing. The same variant has now been recurrently identified as de novo and functionally characterised in the study of Bott et al. [14]. The variant could be classified as pathogenic with PS2_VSTR, PS3_SUP, PS4_MOD, PM2_SUP and PP2, further supplementing hypotonia and macrosomia as possible manifestations in *ATP6V0A1*-related disorders.

#### 3.1.3. *CNTN2*

Contactin-2 is a neuronal membrane protein contributing to the organisation of axonal domains, and variants in the corresponding gene *CNTN2* (HGNC:2172, MIM *190197) have been associated with autosomal recessive myoclonic epilepsy [16]. The phenotypic association is, however, still provisional in OMIM, and currently only the initial study by Stogmann et al. [16] and one case report are available [17].

In a 13 years-old boy presenting with an epileptic encephalopathy and global developmental delay, we identified a homozygous stop-gaining variant in *CNTN2* (NM_005076.4:c.940C>T, p.(Arg314*)). This variant has not been reported in the general population (gnomAD), nor in variant databases. In a subsequent segregation analysis, this variant could be identified in a homozygous state in the similarly affected brother and in a heterozygous state in the unaffected sister, mother and father, confirming the autosomal recessive mode of inheritance. The variant could be classified as pathogenic with PVS1, PM3_SUP and PM2_SUP. Given the growing evidence for the gene–disease relationship, we consider the phenotypic association of *CNTN2* with autosomal recessive myoclonic epilepsy as validated.

#### 3.1.4. *GABRD*

The first association of variants in *GABRD* (HGNC:4084) with generalised epilepsy was proposed 18 years ago by Dibbens et al. [18], who identified two families with idiopathic generalised epilepsy and potential variants in *GABRD*. Only a few independent studies could further confirm this association, which is why the OMIM association is still provisional (MIM #613060).

In a 36 years-old woman presenting with generalised non-motor seizures since the age of two years and mild intellectual disability, we identified the heterozygous variant NM_000815.4:c.872C>T, p.(Thr291Ile), which is not present in the general population, located in a neurotransmitter-gated ion-channel domain and in silico predicted to be pathogenic. Segregation analyses confirmed this variant as being de novo. The affected woman is a mother of monozygotic twins, which were 9 years old at the time of evaluation. Both likewise presented with idiopathic generalised seizures, mild developmental delay and mild intellectual disability. In both affected sons, the abovementioned variant could be identified in a subsequent segregation analysis. The de novo occurrence and the inheritance pattern as well as the in silico parameters strongly suggest pathogenic variants in *GABRD* as causative agents for generalised seizures. The woman and her two sons could be included in the recently published cohort of Ahring et al. [19].

### 3.2. Genes with a Clear Broadening of the Phenotype

#### 3.2.1. *YAP1*

The association of heterozygous loss-of-function variants in *YAP1* (HGNC:16262) and ocular coloboma (MIM #120433) was initially described by Williamson et al. [20]. In 13 individuals, they detected nonsense variants and proposed a reduced penetrance, as some individuals harbouring these variants were apparently not affected. Interestingly, six individuals also presented with learning disabilities, indicating an association with a mild form of intellectual disability. Two further studies provided evidence for the association with ocular colobomas, including an individual with autistic behaviour [21,22].

In an 18-month-old boy with bilateral iris coloboma, staphyloma, morning glory disc anomaly, macular atrophy, microcephaly, hypotonia, dysplastic auricles and developmental delay, we identified the heterozygous frameshift variant NM_001130145.2:c.1196_1199del, p.(Asp399Valfs*3) in the *YAP1*-gene. In his similarly affected 6 years-old brother, the same variant could be identified. Segregation analyses with the parents, however, revealed the variant to be inherited from the unaffected father, undermining the proposed reduced penetrance. Although the symptoms of the two brothers rather represent the severe end of the phenotypic spectrum, the overlap confirms an association of *YAP1* not only with ocular anomalies, but also with intellectual disability and developmental delay.

#### 3.2.2. *CC2D1A*

Pathogenic variants in *CC2D1A* (HGNC:30237) are associated with a recessive form of mental retardation (MIM #608443). The first study describing this association identified affected individuals with intellectual disability without syndromic stigmata [23]. In addition, in a follow-up study analysing additional individuals, the authors could not identify dysmorphic features [24].

Here, we present a 2 years-old girl with global developmental delay, microphthalmia, aniridia, corneal opacity and renal agenesis. Single exome sequencing revealed two heterozygous variants in the *CC2D1A*-gene, which were confirmed to be in trans in a subsequent segregation analysis: NM_017721.4:c.1620_1623dup, p.(Pro542Alafs*38), paternally inherited, and NM_017721.4:c.1345G>A, p.(Val449Met), maternally inherited. To exclude a further potential genetic cause, a Trio-Exome analysis was performed, which revealed no clinically relevant variants apart from the abovementioned compound heterozygous variants. As the number of available studies is limited and no association with ocular or renal anomalies has been described so far, we propose the expansion of the phenotypic spectrum of *CC2D1A*-related disorders to discard the non-syndromic limitation.

#### 3.2.3. *KCTD17*

*KCTD17* (HGNC:25705) has already been described as an established gene associated with myoclonic dystonia (MIM #616398). Affected individuals present with progressive movement disorders, and interestingly, in some individuals, psychiatric abnormalities can also be observed [25]. While the initial study identified one missense variant as being responsible for the phenotype in two families, two recent studies identified pathogenic splicing variants, suggesting haploinsufficiency as the causative mechanism [26,27]. The individuals in these studies presented with childhood-onset dystonia; additionally, the affected girl described by Graziola et al. [27] exhibited a motor delay and normal-to-lower cognitive abilities, which were hypothesised as additional clinical features.

Here, we report a 32 years-old woman with a global developmental delay, generalised dystonia and intellectual disability. She had difficulties sitting at the age of seven months, was able to walk independently at the age of 2 years and showed delayed speech acquisition at the age of 6 years. The generalised dystonia was accompanied by intermittent orofacial dyskinesia and dysarthria. Her mother exhibited similar, but milder dystonia and mild developmental delay in childhood. Via exome sequencing, we identified the heterozygous, likely pathogenic frameshift variant NM_001282684.1:c.557_558del, p.(Glu186Valfs*67). Trio-Exome sequencing was performed subsequently to segregate the identified variant and to exclude a second genetic cause, as the association of this gene with developmental delay and intellectual disability was not well-established. The variant was indeed inherited from the mother, and no further candidate variant was identified in the trio setting. These results not only expand the phenotypic spectrum by adding developmental disorders and intellectual disabilities as possible symptoms, but also confirm haploinsufficiency as a causative mechanism in *KCTD17*-associated diseases.

### 3.3. Genes with Insufficient Evidence in the Medical Literature

#### 3.3.1. *RUSC2*

Biallelic truncating variants in *RUSC2* (HGNC:23625) are associated with an autosomal recessive intellectual developmental disorder (MIM #617773). The initial study by Alwadei et al. [28] described two affected brothers and an unrelated girl with hypotonia, intellectual disability, seizures and secondary microcephaly, in whom they identified homozygous inherited loss-of-function variants. Until now, some additional pathogenic variants have been submitted to ClinVar, yet additional case reports validating the phenotypic association for this gene are lacking. The OMIM entry is exclusively based on the abovementioned study, which proposed loss of function as the pathomechanism. However, many missense variants of uncertain significance in ClinVar were identified in individuals potentially affected with an *RUSC2*-associated disorder, but no pathogenic missense variant has been identified yet. Therefore, the interpretation of variants not directly linked to a loss-of-function mechanism remains challenging.

Here we report a 31 years-old man with severe intellectual disability, bilateral tonic–clonic seizure with focal onset, autism, obesity and insomnia. Exome sequencing revealed two variants in the *RUSC2*-gene (NM_001135999.1): c.1825C>T, p.(Leu609Phe) and c.3235+2T>A, p.?. Subsequent segregation analysis determined these variants in trans and as being maternally and paternally inherited, respectively. Both variants present a very low allele count in the general population, and the splice variant is expected to result in an out-of-frame exon skipping of exon 7, which could cause a non-functioning shortened protein or degradation via nonsense-mediated decay. Given that hitherto only biallelic loss-of-function variants have been described as disease-causing in *RUSC2*, the interpretation of this identified missense variant and the variants of uncertain significance in ClinVar is hampered. However, this disease mechanism has, as mentioned above, until now only been suggested in the initial study of Alwadei et al. [28]. Further studies, especially functional analyses of missense variants, are needed to facilitate the confirmation of the disease mechanism.

#### 3.3.2. *PLAG1*

Pathogenic variants in *PLAG1* (HGNC:9045) have been associated with a novel form of Silver–Russel syndrome (MIM #618907). Until now, five studies have confirmed this link [29,30,31,32,33]. In a 23 years-old male individual presenting with congenital short stature, we were able to identify the heterozygous variant NM_002655.3:c.758dup, p.(Phe254Ilefs*23). Segregation analyses with Sanger sequencing revealed this variant to be de novo, resulting in a classification as likely pathogenic with PVS1_STR, PS2_SUP and PM2_SUP, thus further confirming the association of *PLAG1* with a form of Silver–Russel syndrome.

#### 3.3.3. *AP2M1*

The association between *AP2M1* (HGNC:564) and intellectual developmental disorder with seizures (MIM #618587) has hitherto been based on a single study performed by Helbig et al. [34]. In four unrelated individuals, they identified the same recurrent variant, NM_004068.4:c.508C>T, p.(Arg170Trp). Based on functional analyses, the authors concluded that this amino acid position was of particular relevance for clathrin-mediated endocytosis and thus as a disease mechanism for neurodevelopmental disorders. Variants in other amino acid positions, however, have not been ruled out as being of similar clinical relevance, and, to our knowledge, no other pathogenic variants in this gene have been identified yet.

In a 3 years-old boy presenting with global developmental delay, intellectual disability and macrocephaly, we identified the heterozygous de novo variant NM_004068.4:c.73G>A, p.(Gly25Arg), which was not present in the general population and is situated in the clathrin adaptor complex small chain domain. In contrast to the individuals described by Helbig et al. [34], the boy did not present with seizures, but EEG analyses showed pathologic multifocal epileptiform activity. The preceding chromosome analysis revealed a translocation of the short arm of chromosome 11 and the long arm of chromosome 17: t(11;17)(p13;q23.3). However, NGS-based CNV analysis could not identify a disruption of the genes in these regions, and segregation analyses showed this translocation to be maternally inherited. We thus interpreted this translocation as being clinically irrelevant and considered the case solved based on the de novo variant in *AP2M1*, expanding the number of causative variants in this gene.

## 4. Discussion

Our comprehensive in-house dataset revealed that we had reported 77 genes with limited evidence in public databases to the referring physicians. Such genes with low GDAs raise the diagnostic yield and enrich the literature when the interpretation of their relevance is correct, but they are also a risk for false-positive reports if interpreted falsely.

Retrospective analyses showed that 35 of these genes were already present with sufficient evidence in the medical literature, even though the number of distinct pathogenic variants in the public databases was limited. There are several reasons for this pattern: Some genes are well-characterised, even if they are based on only a few cases. This might be due to highly specific phenotypes (e.g., *AP2S1* [35]), well-established functional studies (e.g., *CFAP52* [36]) or broad phenotypic characterisation of the published individuals (e.g., *MDH2* [37]). Some genes, such as *PACS1* and *PACS2*, exhibit only single but recurrent variants that are causative with respect to specific disorders, which are then, however, well-discriminated.

While routine work generates an ever-increasing number of carefully weighted findings, many of them are not available outside of the institutions they were generated in. Variants from published case reports or cohorts are not always submitted to ClinVar and can only be retrieved by HGMD. While HGMD automatically includes variants from screened literature, variants in ClinVar have to be actively submitted. For some genes, this leads to a shift in available pathogenic variants when both databases are compared (Figure 1). This also highlights the necessity of screening several databases for a final validation of a gene with supposedly limited evidence. We assume that uploading variants to ClinVar is feasible for every lab. Our impression is that diagnostic labs overestimate the time and effort needed and, unfortunately, refrain from doing so. ClinVar provides a comprehensive and freely available overview of clinically relevant variants, and we encourage clinicians and researchers to regularly submit their identified variants and associated symptoms for greater accessibility across institutes. Providing consistent evidence on rare genes is crucial for a reliable and final diagnosis, and new evidence can provide sufficient information for an uncertain case to be considered solved (Box 1).

On the contrary, reporting genes with limited evidence can also lead to false-positive results. Here, nine of the abovementioned 77 genes turned out to be irrelevant after retrospective analysis (Supplementary Table S1). For example, a variant in *TMLHE* could not be confirmed after segregation analyses, as the unaffected father carried the same variant and the similarly affected sister did not. In addition, the variant in *NSMF* turned out to be irrelevant, as a more convincing deletion of 41 genes could be identified in a subsequent analysis. While we in general recommend reporting rare or even unusual variants, these nine cases strongly highlight the need for regular re-evaluations in routine diagnostics, not only to confirm the proposed genetic cause communicated to the affected individual, but also to retract variants that turn out to be irrelevant [38]. There are several types of re-evaluations. One type would be the bulk re-analysis of all negative cases in order to find potentially missed variants. Note that re-sequencing of whole datasets is not necessarily needed, as a re-annotation can already have a high impact and unravel new relevant variants [39]. Another type would be the re-evaluation of possibly clarified cases with respect to some uncertainty, in order to determine whether the reported genes and variants are still valid. We think that the latter type of re-evaluation is feasible for every lab. While we do not consider it necessary to re-check every ever-reported positive case, we do believe this can be useful for those with less valid variants and genes. Usually, every reporter is aware of the strength of the reported variant, and such cases can be tagged and re-evaluated later. It is also possible to tag potentially relevant variants that are not reported, which can be followed up in subsequent re-evaluations later (Box 1). A further seven genes remained open regarding their relevance, as we did not have access to more information or material to confirm or retract the diagnosis. Some genes harboured splice variants outside the canonical splice sites. Correctly determining their impacts on the protein products—including alternative splicing patterns in lowly expressed transcripts—remains challenging. Exome sequencing usually reveals splice variants some hundred base pairs up- and downstream of exon boundaries, but functional assessments of novel variants can hardly be performed in a diagnostic setting. Identification and characterisation of splice variants in research settings are therefore needed to expand the databases, aiding the interpretation of identified variants in routine diagnostics.

For the remaining 21 genes, our results broaden the phenotypic spectrum, substantially strengthen the confirmation of genes with limited evidence in the medical literature or confirm lacking or provisional OMIM associations. The series of case reports that we show in the results also aim to validate these missing OMIM entries and expand the spectrum of potential symptoms and disease mechanisms. Increasingly, clinicians and researchers will have to deal with newly described genes harbouring reduced penetrances, such as *YAP1*. In addition, the phenotypic variability remains challenging, as variants in genes such as *KCTD17* may or may not lead to developmental disorders. It is therefore of particular importance to communicate potential phenotypic manifestations and penetrance patterns in public databases or via updated OMIM entries to facilitate the reliable diagnosis of affected individuals.

Short case reports are useful tools to provide additional evidence for newly described genes and do not require tremendous research efforts. Still, we are aware that even such moderate research efforts are not feasible in the settings of many diagnostic labs. An alternative would be to upload the variants to ClinVar with more information (e.g., including HPO terms) or upload the variants to matchmaking platforms or engage in cooperation with a research lab that may undertake such efforts (Box 1). Our experience shows that all three of the abovementioned recommendations are good alternatives to the cumbersome writing of manuscripts.

Another challenge is the burden of classifying variants as (likely) pathogenic. The ACMG criteria provide a standardised framework for variant classification, which is also considered best practice in clinical genetics. However, a variant can also be considered as causative even though the formal criteria only indicate uncertain significance. While we in general support the usage of standardised criteria, it remains challenging to communicate the final conclusion for each individual in public databases. An example here would be our identified variant in *NCKAP1*. The formal classification based on these criteria is a variant of uncertain significance. However, the pathomechanism, phenotypic overlap and de novo occurrence provide sufficient evidence for the case to be considered solved, which can, however, not be reflected in public databases, such as ClinVar. This is also true for our variants detected in *AP2M1* and *RUSC2*, which expand the number of causative variants and/or disease mechanisms. Additionally, this undermines the importance of, e.g., case reports, where other attributes apart from the formal classification can provide additional evidence for a newly described gene as being of clinical relevance. As Figure 1 illustrates, especially genes associated with neurodevelopmental delay and/or epilepsy have limited evidence, indicating a continuously evolving field of research.

Identification of rare variants in routine diagnostics can also facilitate research through the sharing of these results via matchmaking databases, such as GeneMatcher [40]. With this approach, several individuals from our cohort could be included in larger studies, such as *GNAI1* [41], *RIPPLY2* [42] and *EIF3F* [43]. Further case reports or the inclusion of our affected individual in larger cohorts are planned for *RAB11B*, *RHEB*, *ADARB1*, *GABRB1* and *TCF7L2* to provide additional support for weak GDAs, expand the phenotypic spectrum or validate OMIM phenotypes.

A possible limitation of this study is the bias of reporters. Cases in which reporters refrained from reporting an insecure variant based on subjective perceptions were not retrospectively evaluated here. Variants in genes with weak evidence may, however, be discovered with the accumulation of knowledge after subsequent re-analysis. One further limitation of this study is that we are aware that we are an academic institute with an interest in research. This is not the case for every diagnostic lab. As outlined in Box 1, we nevertheless have tried to make suggestions that are practicable for every lab. In our view, these recommendations are feasible for all labs, regardless of setting (e.g., academic or private) or performance in terms of scientific impact.

Box 1 **Recommendations for diagnostic laboratories on handling and communicating rare genes and variants to the scientific community.**

1. Upload all identified variants, the associated phenotypes/HPO terms and the applied ACMG classification criteria to public databases, such as ClinVar. For variants of uncertain significance, indicate whether the case is considered solved based on the variant in question using the comment section in ClinVar.
2. Perform regular re-evaluations and tag identified variants and genes with limited evidence for re-evaluation. There is no need to re-evaluate genes and variants with strong evidence.
3. Write short case reports or compact cohorts with descriptions of all identified variants and phenotypes. If this is not feasible, upload to ClinVar or to matchmaking platforms, such as GeneMatcher, to facilitate research efforts in other institutes.

## 5. Conclusions

Through routine diagnostics, we have confirmed and delineated 21 genes as being truly associated with neurodevelopmental disorders. We found that reporting genes with weak GDAs to physicians carries the risk of false-positive results on the one hand, while on the other hand re-evaluations can provide substantial support for novel disorders. Taken together, we encourage clinicians and scientists in routine diagnostics to regularly submit identified variants and associated symptoms to public databases and to provide additional evidence through regular case reports.

## Figures and Tables

**Table 1:** Criteria for the consideration of a case report. We considered a case report to be worthwhile if it confirms GDAs for genes lacking OMIM entries, broadens the phenotypic spectrum or strengthens the GDA for newly described disorders. A case report would be unattractive if there were already sufficient evidence on the GDA, the information on the pathomechanism were insufficient, the patient and/or the parents were not available for further analyses or if the gene turned out to be irrelevant.

Report Worthwhile	Criteria	Description
Yes	Confirms proposed GDA lacking an OMIM entry	Genes that lack an OMIM entry or only have provisional associations are confirmed here
	Broadens phenotypic spectrum	Provides additional symptoms of described disorders
	Strengthens GDA for novel disorders	Confirms phenotype for genes that may only be described based on a few patients
No	Sufficient evidence	Recent cohorts and/or case reports already provide enough evidence for this GDA
	Insufficient information on pathomechanism	For example, identified loss-of-function variants in genes where only missense variants are described without clear evidence on haploinsufficiency as the pathomechanism
	Patient and/or parents not available	Patient deceased and is not available for further analyses, or parents are not available for segregation analyses
	Irrelevant	Re-evaluation identified more convincing variants, or the proposed phenotype could not be confirmed

**Table 2:** Genes with worthwhile case reports identified in routine diagnostics. Genes with detailed descriptions in this publication are labelled with “see main text”. For more information, see Supplementary Table S1.

Gene	Variant (Zygosity)	Origin	Classification	Disease Group	OMIM Phenotype (ID)	Report Worthwhile	Comment
*ADARB1*	NM_015833.4:c.1299dup, p.(Phe434Valfs*2) (homo)	Paternal and maternal	VUS	NDD + Epilepsy	Neurodevelopmental disorder with hypotonia, microcephaly and seizures (#618862)	Yes, strengthens GDA	Is going to be published separately
*AP2M1*	NM_001311198.2:c.73G>A, p.(Gly25Arg) (het)	De novo	VUS	NDD	Intellectual developmental disorder 60 with seizures (#618587)	Yes, strengthens GDA	See main text; first validation of genotype-phenotype association
*ATP6V0A1*	NM_001130020.3:c.2222G>A, p.(Arg741Gln) (het)	Not maternal	Likely pathogenic	NDD + Epilepsy	NA	Yes, confirms GDA lacking OMIM entry	See main text; our patient confirms the proposed phenotype in the literature, which was lacking an OMIM entry
*BCKDK*	NM_005881.4:c.50_71del, p.(Leu17Hisfs*47) (homo)	NA	Pathogenic	NDD + Epilepsy	Branched-chain ketoacid dehydrogenase kinase deficiency (#248600)	Yes, strengthens GDA	Further support for the phenotype hitherto only supported by few studies
*BCORL1*	NM_001184772.3:c.3403C>T, p.(Gln1135*) (hemi)	Maternal	Likely pathogenic	NDD + Epilepsy	Shukla–Vernon syndrome (#301029)	Yes, strengthens GDA	Further support for the phenotype hitherto only supported by few studies
*CARS2*	NM_024537.4:c.649_651del, p.(Glu217del) (homo)	NA	Likely pathogenic	NDD + Epilepsy	Combined oxidative phosphorylation deficiency 27 (#616672)	Yes, strengthens GDA	Further support for the phenotype hitherto only supported by few studies
*CC2D1A*	NM_017721.5:c.1620_1623dup, p.(Pro542Alafs*38); c.1345G>A, p.(Val449Met) (comphet)	Maternal and paternal	Likely pathogenic	NDD	Mental retardation, autosomal recessive 3 (#608443)	Yes, broadens phenotypic spectrum	See main text; our patient adds syndromic stigmata and ocular anomalies to the phenotypic spectrum
*CNTN2*	NM_005076.5:c.940C>T, p.(Arg314*) (homo)	Paternal and maternal	Likely pathogenic	NDD + Epilepsy	NA	Yes, confirms GDA lacking OMIM entry	See main text; our patient confirms the hitherto provisional OMIM association
*FBXO38*	NM_030793.5:c.1313A>G, p.(His438Arg) (het)	NA	VUS	Neuro	Neuronopathy, distal hereditary motor, type IID (#615575)	Yes, strengthens GDA	Many VUSs, only two pathogenic variants found
*GABRB1*	NM_000812.4:c.854C>A, p.(Thr285Lys) (het)	De novo	Likely pathogenic	NDD + Epilepsy	Developmental and epileptic encephalopathy 45 (#617153)	Yes, strengthens GDA	Is going to be published separately, patient is included in a larger cohort
*GABRB1*	NM_000812.4:c.860C>T, p.(Thr287Ile) (het)	NA	Likely pathogenic	NDD + Epilepsy	Developmental and epileptic encephalopathy 45 (#617153)	Yes, strengthens GDA	Is going to be published separately, patient is included in a larger cohort
*GABRB1*	NM_000812.4:c.757C>T, p.(Pro253Ser) (het)	De novo	Likely pathogenic	Epilepsy	Developmental and epileptic encephalopathy 45 (#617153)	Yes, strengthens GDA	Is going to be published separately, patient is included in a larger cohort
*GABRD*	NM_000815.5:c.872C>T, p.(Thr291Ile) (het)	De novo	VUS	NDD + Epilepsy	NA	Yes, confirms GDA lacking OMIM entry	See main text; our patient confirms the proposed phenotype in the literature, which was lacking an OMIM entry
*KAT8*	NM_182958.4:c.524A>C, p.(Lys175Thr) (het)	De novo	VUS	NDD	Li–Ghorgani–Weisz–Hubshman syndrome (#618974)	Yes, strengthens GDA	Subsequent segregation analyses determined this variant to be de novo
*KCTD17*	NM_001282684.1:c.557_558del, p.(Glu186Valfs*67) (het)	Maternal	Likely pathogenic	NDD	Dystonia 26, myoclonic (#616398)	Yes, broadens phenotypic spectrum	See main text; our patient adds intellectual disability to the phenotypic spectrum
*NCKAP1*	NM_205842.3:c.3366_3369del, p.(Tyr1122fs) (het)	De novo	VUS	NDD + Epilepsy	NA	Yes, confirms GDA lacking OMIM entry	See main text; is also going to be published separately, patient is included in a larger cohort
*PRKD1*	NM_001330069.2:c.1754C>T, p.(Pro585Leu) (het)	De novo	VUS	NDD	Congenital heart defects and ectodermal dysplasia (#617364)	Yes, strengthens GDA	Our patient confirms developmental delay and syndromic stigmata of the phenotypic spectrum
*RAB11B*	NM_004218.4:c.97C>T, p.(Arg33Cys) (het)	Not maternal	VUS	NDD	Neurodevelopmental disorder with ataxic gait, absent speech and decreased cortical white matter (#617807)	Yes, strengthens GDA	Is going to be published separately
*RHEB*	NM_005614.4:c.47C>T, p.(Ser16Phe) (het)	De novo	VUS	NDD + Epilepsy	NA	Yes, confirms GDA lacking OMIM entry	Is going to be published separately
*RUSC2*	NM_001135999.1:c.1825C>T, p.(Leu609Phe); c.3235+2T>A, p.? (comphet)	Maternal and paternal	VUS	NDD + Epilepsy	Mental retardation, autosomal recessive 61 (#617773)	Yes, strengthens GDA	See main text; our patient strengthens the proposed recessive inheritance of this disorder
*TCF7L2*	NM_030756.5:c.1187C>T, p.(Ala396Val) (het)	De novo	Likely pathogenic	NDD	NA	Yes, confirms GDA lacking OMIM entry	Is going to be published separately, confirming *TCF7L2* as a gene for syndromic DD via an independent case report
*YAP1*	NM_001130145.3:c.1196_1199del, p.(Asp399Valfs*3) (het)	Paternal	Likely pathogenic	NDD	Coloboma, ocular, with or without hearing impairment, cleft lip/palate and/or mental retardation (#120433)	Yes, broadens phenotypic spectrum	See main text; our patients add developmental delay and syndromic stigmata to the phenotypic spectrum and undermine the proposed reduced penetrance
*ZNF142*	NM_001105537.4:c.1118G>A, p.(Arg373His); c.4091C>G, p.(Pro1364Arg) (comphet)	NA	VUS	NDD + Epilepsy	Neurodevelopmental disorder with impaired speech and hyperkinetic movements (#618425)	Yes, strengthens GDA	Further support for the phenotype hitherto only supported by few studies

## Data Availability

Not applicable.

## Supplementary Materials

The following supporting information can be downloaded at: https://www.mdpi.com/article/10.3390/genes13122305/s1, Supplementary Table S1: Overview of reported genes with limited GDAs.
